# A rat model for retinitis pigmentosa with rapid retinal degeneration enables drug evaluation in vivo

**DOI:** 10.1186/s12575-021-00150-y

**Published:** 2021-06-04

**Authors:** Chisato Inoue, Tamaki Takeuchi, Akira Shiota, Mineo Kondo, Yuji Nshizawa

**Affiliations:** 1 Center for Clinical Examination Practicum Supp, ort, Chubu University, Kasugai, Aichi 4878501 Japan; 2 College of Life and Health Sciences, Chubu University, Kasugai, Aichi 4878501 Japan; 3 Institute of Immunology Co., LTD, Utsunomiya, Tochigi, 3290512 Japan; 4 Department of Ophthalmology, Mie University Graduate School of Medicine, Tsu, Mie 5148507 Japan; 5 Department of Biomedical Sciences, Chubu University, Kasugai, Aichi 4878501 Japan

**Keywords:** Rapamycin, Retina, Photoreceptor, Rat model, Retinitis pigmentosa, Retinal degeneration, Rhodopsin, Apoptosis

## Abstract

**Background:**

Although retinitis pigmentosa (RP) is most frequently studied in mouse models, rats, rabbits, and pigs are also used as animal models of RP. However, no studies have reported postnatal photoreceptor cell loss before complete development in these models. Here, we generated a transgenic rat strain, named the P347L rat, in which proline at position 347 in the rhodopsin protein was replaced with leucine.

**Results:**

A pathological analysis of photoreceptor cells in the P347L rat model was performed, and drugs with potential use as therapeutic agents against RP were investigated. The data clearly showed rapid degeneration and elimination of the outer nuclear layer even before the photoreceptor cells were fully established in P347L rats. To test the usefulness of the P347L rat in the search for new therapeutic agents against RP, the effects of rapamycin on RP were investigated in this rat strain. The findings suggest that rapamycin promotes autophagy and autophagosomal uptake of the rhodopsin that has accumulated abnormally in the cytoplasm, thereby alleviating stress and delaying photoreceptor cell death.

**Conclusions:**

In this RP model, the time to onset of retinal degeneration was less than that of previously reported RP models with other rhodopsin mutations, enabling quicker in vivo evaluation of drug efficacy. Administration of rapamycin delayed the photoreceptor cell degeneration by approximately 1 day.

**Supplementary Information:**

The online version contains supplementary material available at 10.1186/s12575-021-00150-y.

## Background

Retinitis pigmentosa (RP) is a progressive, hereditary eye disease in humans. Approximately 25% of cases of autosomal dominant RP are caused by mutations in the rhodopsin gene [1–3]. Rhodopsin is a G-protein-coupled receptor and is abundant on the outer segments of rod photoreceptor cells [4]. It is thought that rhodopsin plays an important role in the maintenance of rod photoreceptor cell structure. Rhodopsin is synthesized in the inner segment and then transported to the outer segment via the action of cilia. Amino acids at the carboxy-terminus of rhodopsin are involved in this transport [5, 6]. Mutation at the C-terminus can block rhodopsin transport to the outer segment such that the protein accumulates in the cytoplasm, leading to retinal degeneration [7, 8].

Rapamycin is a macrolide antibiotic that is produced by *Streptomyces hygroscopicus*, a soil bacterium of the family Actinomycetaceae that was isolated on Easter Island [9]. Rapamycin has been shown to also have immunosuppressive activity, and therefore, it is now used to prevent organ transplant rejection. Since rapamycin is a specific inhibitor of the mTOR signaling pathway, studies are now being conducted to explore the potential of rapamycin for other therapeutic effects [10]. These studies have shown that rapamycin is neuroprotective in various neurodegenerative diseases [11]. In addition, in vitro studies using induced pluripotent stem cells have suggested that rapamycin can be used as a therapeutic agent against RP [12].

In the present study, we aimed to determine the protective effects of rapamycin against retinal degeneration, and to this end, we performed a morphological evaluation of these effects in vivo using a newly developed RP rat model.

## Results

### Characterization of the P347L Transgenic Rat Model

#### Rapid postnatal retinal degeneration in P347L rats

**Fig. 1:**
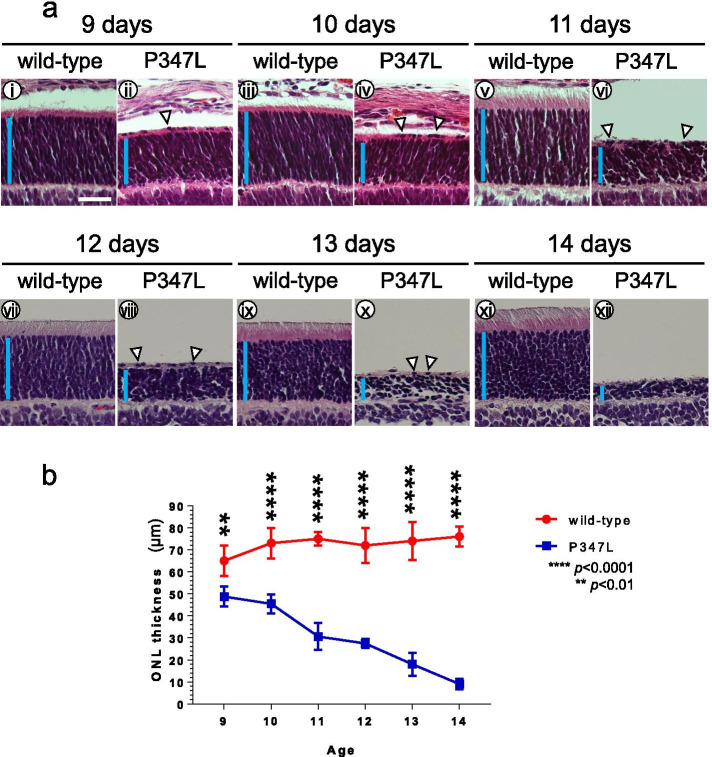
Retinal degeneration in P347L rats. (a) The retina was analyzed in P347L rats from the ages of 9 to 14 days by hematoxylin–eosin staining. In wild-type rats, the outer nuclear layer (ONL) was fully formed at the age of 9 days (i), and the inner and outer segments expanded up to the age of 14 days (xi). In P347L rats, the ONL decreased from the age of 9 days (ii), and this layer was no longer discernible at 14 days (xii). Heterotopic cells were found in the inner segment in the ONL of P347L rats. Retinas were examined at the age of 9 days (i, ii), 10 days (iii, iv); 11 days (v, vi); 12 days (vii, viii); 13 days (ix, x); and 14 days (xi, xii). Arrowheads: heterotopic cells; scale bar: 30 μm; vertical bar: ONL. (b) Graph showing the outer nuclear thickness. Statistical analysis was performed by two-way ANOVA with Sidak’s multiple comparisons test: **p < 0.01; ****p < 0.0001

To investigate degeneration of the retinal photoreceptor layer in P347L rats, retinas of wild-type and P347L rats were analyzed continuously by hematoxylin–eosin (HE) staining from the ages of 9 to 14 days. In wild-type rats, the outer nuclear layers (ONLs) of photoreceptor cells were fully formed by 9 days, and the inner and outer segments expanded until 14 days (Fig. 1a-i, iii, v, vii, ix, xi). In contrast, the ONL began to decrease by 9 days in P347L rats, and by 14 days most of the photoreceptor cells were denatured or eliminated (Fig. 1a-ii, iv, vi, viii, x, xii, b). In addition, heterotopic cells were found in the retinas of P347L rats. These data clearly show that rapid degeneration and elimination of the ONL occurred even before the photoreceptor cells were fully established in P347L rats.

### Rhodopsin accumulation in P347L rat photoreceptor cells

**Fig. 2:**
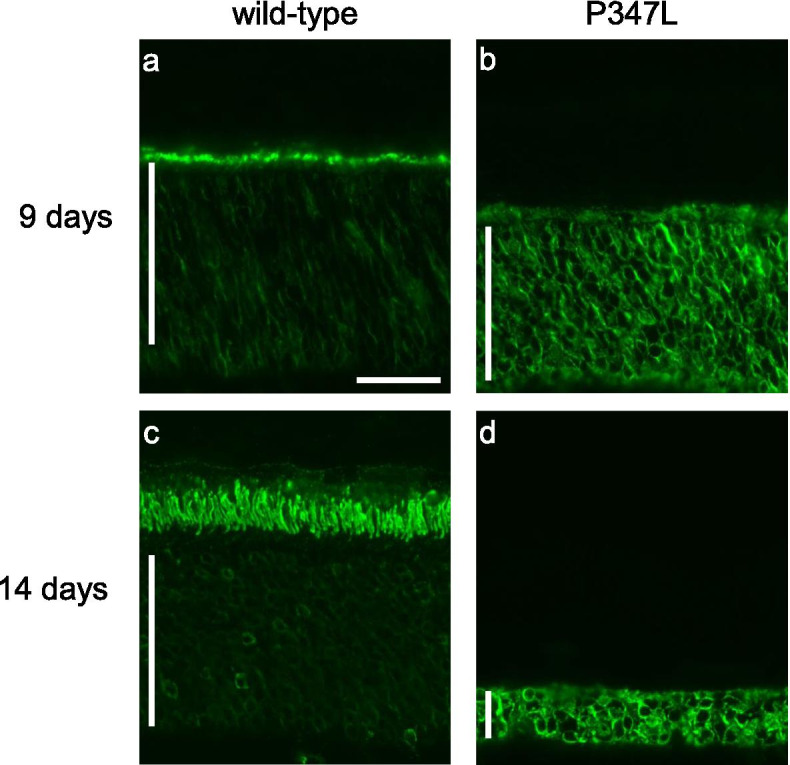
Rhodopsin immunolocalization in the retinal outer nuclear layer (ONL). Fluorescent immunostaining using rhodopsin antibodies showing the localization of rhodopsin in the retinas of wild-type and P347L rats at the age of 9 days (**a**, **b**) and 14 days (**c**, **d**). Scale bar: 30 μm, vertical bar: ONL

To investigate the localization of rhodopsin in the retinal photoreceptor ONL, fluorescent antibody staining was performed using rhodopsin antibodies. At the age of 9 days, retinas of wild-type rats were intensely stained, indicating that rhodopsin was synthesized in the ONL and transported to the inner segment (Fig. 2a). In P347L rats, rhodopsin accumulated in the photoreceptor cytoplasm by the age of 9 days (Fig. 2b). Moreover, retinas of 14-day-old wild-type rats exhibited intense staining for rhodopsin (Fig. 2c), showing fully formed outer segments. In P347L rats, in contrast, the cytoplasm showed staining for rhodopsin, indicating that the photoreceptor cells had degenerated and decreased in number.

### Copy number analysis of P347L rat genome

When the copy number polymorphism in the P347L rat genome was examined by a TaqMan® Copy number assay, approximately 9 copies of the transgene were found to be inserted in the genome (Supplemental Fig. 2).

### Promotion of ER stress response and autophagy in P347L rat photoreceptor cells

**Fig. 3:**
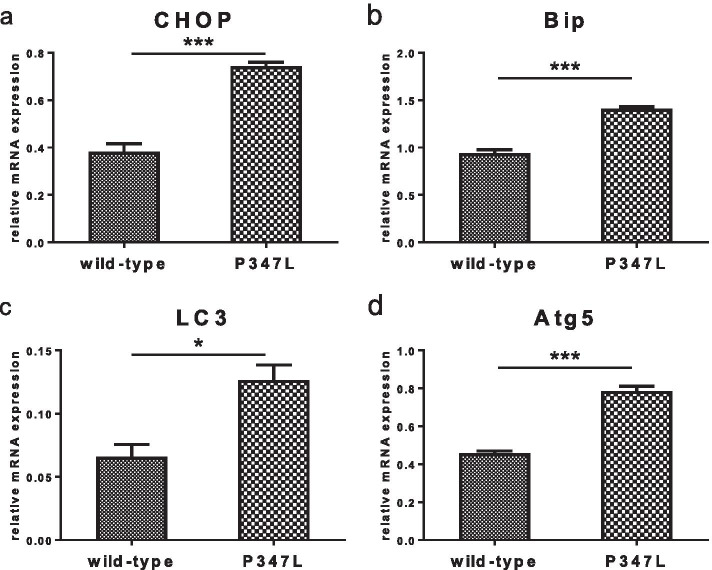
mRNA expression analysis. mRNA expression levels of various genes in retinas of the wildtype and P347L rats at the age of 10 days were analyzed and compared by real-time PCR. mRNA expression levels of ER stress markers CHOP and Bip were significantly higher in P347L rats than in wild-type rats (**a**, **b**). mRNA expression levels of autophagy related molecules LC3 and Atg5 were significantly increased in P347L rats (**c**, **d**). Unpaired *t* test: *p < 0.05; ***p < 0.001. n = 3

Mutations at the C-terminus of rhodopsin block its transport to the outer segment, leading to cytoplasmic accumulation; therefore, we investigated the association of the P347L mutation with ER stress and autophagy. Using quantitative reverse-transcription polymerase chain reaction (qRT-PCR), the expression levels of ER stress markers CHOP and Bip were compared and found to be significantly higher in P347L rats than in wild-type rats (Fig. 3). In addition, the expression levels of autophagy markers LC3 and Atg5 were found to be significantly higher in P347L rats than in wild-type rats (Fig. 3).

### Analysis of morphological changes in photoreceptor cells following drug administration

#### Transient suppression of P347L rat retinal degeneration by rapamycin

**Fig. 4:**
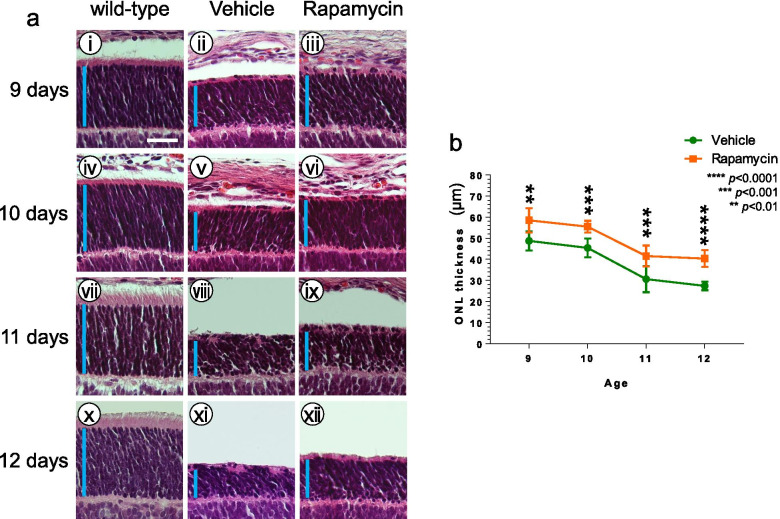
Effects of rapamycin administration on retinal degeneration. (a) Changes in the thickness of the retinal outer nuclear layer (ONL) were assessed continuously by hematoxylin–eosin staining at the ages of 9 to 12 days, after intravitreal administration of rapamycin at the age of 8 days. Wild-type rat retinas are shown in panels i, iv, vii, and x. Compared to the vehicle group, the loss of the ONL was suppressed in the rapamycin group (ii versus iii; v versus vi; viii versus ix; xi versus xii). Scale bar: 30 μm; vertical bar: ONL. (b) Graph showing the ONL thickness in P347L rats with or without rapamycin. Statistical analysis was performed by two-way ANOVA with Sidak’s multiple-comparisons test: ****p < 0.0001; ***p < 0.001; **p < 0.01

To examine the effects of rapamycin on retinal degeneration, rapamycin was administered to P347L rats by intravitreal injection. Retinas were collected at the age of 9 to 12 days, and HE staining was performed. The thickness of the ONL was measured at a site that was intermediate between the corneal tip and the optic nerve papilla but more proximal to the papilla. At this point, the inner nuclear layer had a fixed number of cells. At 9 to 12 days, the ONL loss was suppressed in rapamycin-treated rats (rapamycin group) compared to that of the vehicle group (Fig. 4a, b). However, the rapamycin group subsequently showed loss of the ONL, indicating that the suppressive effects of the drug were transient.

**Fig. 5:**
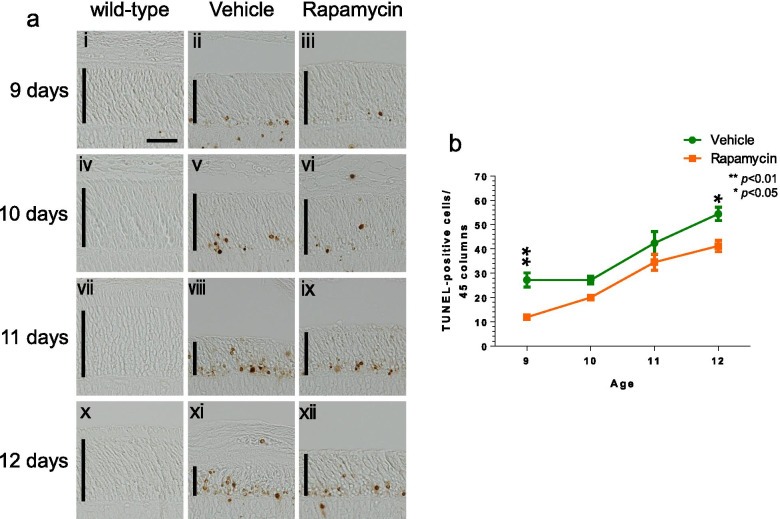
Effects of rapamycin administration on retinal degeneration. (a) Apoptosis-positive cells in the outer nuclear layer (ONL) were analyzed continuously by TUNEL staining at the ages of 9 to 12 days, after intravitreal administration of rapamycin at the age of 8 days. Wild-type rat retinas are shown in panels a- i, iv, vii, and x. Compared to the vehicle group the number of apoptosis-positive cells in the rapamycin group is small in P347L rats; 45% versus 75% (ii versus iii; v versus vi; viii versus ix; xi versus xii). Scale bar: 30 μm; vertical bar: ONL. (b) Graphical representation of the number of TUNEL positive cells per 45 columns of photoreceptor cells. The number of apoptosis-positive cells was significantly reduced at the ages of 9 to 12 days. Statistical analysis was performed by two-way ANOVA with Sidak’s multiple-comparison test: **p < 0.01; *p < 0.05

Additionally, retinal TUNEL staining showed changes in the number of apoptosis-positive cells. At each stage, a comparison of TUNEL staining images between the vehicle and rapamycin groups revealed fewer apoptosis-positive cells in the rapamycin group (Fig. 5a). Quantification of the results indicated that the number of apoptosis-positive cells per 45 columns of photoreceptor cells was lower in the rapamycin group than in the vehicle group at the ages of 9 to 12 days (Fig. 5b). Notably, rapamycin administration delayed photoreceptor cell degeneration by approximately 1 day (Fig. 5b).

### Induction of P347L rat retinal degeneration through the caspase-3-dependent pathway

**Fig. 6:**
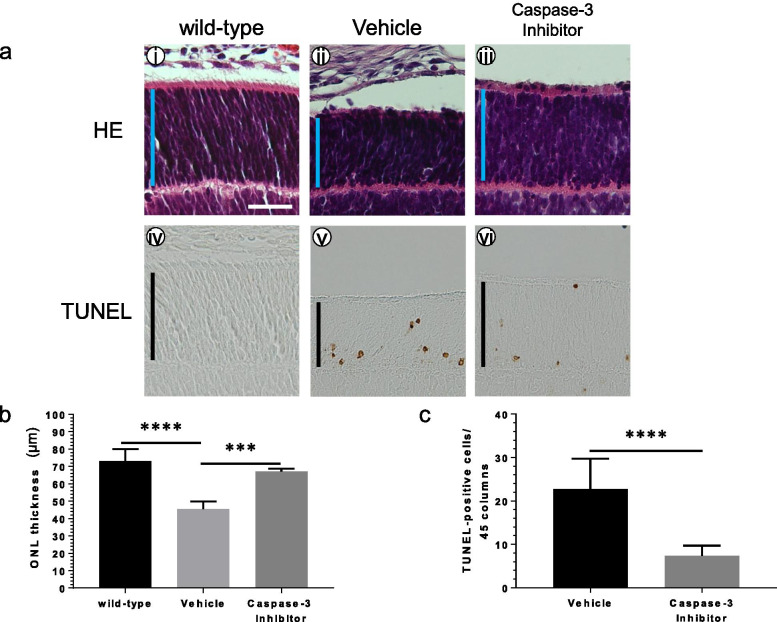
Morphological changes in retinas due to administration of a caspase-3 inhibitor. (a) Analysis of retinas after intravitreal administration of a caspase-3 inhibitor using hematoxylin–eosin (i-iii) at the age of 10 days and TUNEL staining (iv-vi) at the age of 10 days. Wild-type rat retinas are shown in panels (i) and (iv). The loss of the outer nuclear layer (ONL) was delayed in the caspase-3 inhibitor group (iii) compared with the vehicle group (ii). The number of apoptosis-positive cells was clearly less in the caspase-3 inhibitor group (vi) relative to the vehicle group (v). Scale bar: 30 μm; vertical bar: ONL. (b) Graph showing the ONL thickness at the age of 10 days. Statistical analysis was performed by one-way ANOVA with Tukey’s multiple comparisons test: ***p < 0.001; ****p < 0.0001. (c) Graphical representation of the number of TUNEL positive cells per 45 columns of photoreceptor cells. The number of apoptosis-positive cells was significantly reduced at the age of 10 days. Unpaired *t* test: ****p < 0.0001

To gain further insights into retinal degeneration in P347L rats, a caspase-3 inhibitor was administered via intravitreal injection, and morphological changes in the retinal ONL were evaluated using HE staining at the age of 10 days. Two days after drug administration, ONL loss was suppressed in the caspase-3 inhibitor group compared to that in the vehicle group (Fig. 6a-ii, iii, b). TUNEL staining showed that the number of apoptosis-positive cells in the caspase-3 inhibitor group was much lower than that in the vehicle group (Fig. 6a-v, vi, c). Relative to untreated P347L rats, the number of apoptosis-positive cells in the caspase-3 inhibitor treated P347L rats was very small, and the thickness of the ONL was scarcely decreased (Fig. 5a-vi). The marked suppression of apoptosis due to inhibition of caspase-3 shows that retinal degeneration in P347L rats primarily involves apoptosis induced via a caspase-dependent pathway.

### Increase in autophagosomal rhodopsin uptake due to rapamycin

**Fig. 7:**
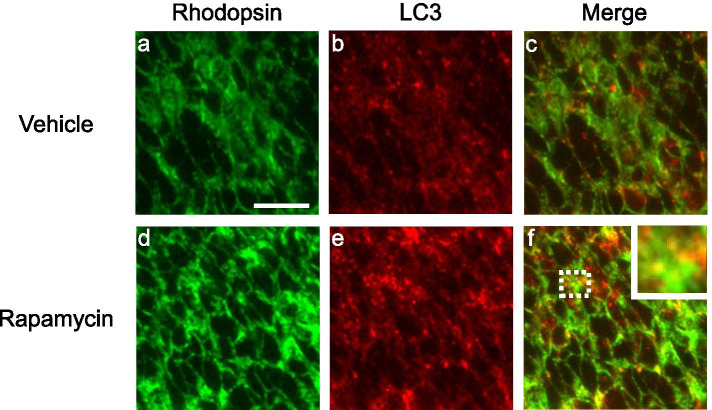
Rhodopsin and LC3 localization in retinal outer nuclear layer (ONL) after rapamycin administration. Fluorescence imaging of retinas for the expression of rhodopsin and LC3 in the vehicle control group (**a**, **b**) and rapamycin treated group (**d**, **e**). Superimposed images showing co-localization of rhodopsin and LC3 (**c**, **f**). Inset is an enlargement of the part surrounded by the dotted line. Scale bar: 50 μm

At the age of 8 days, rapamycin was administered intravitreally, and the retina was examined 2 days post-treatment. Fluorescent antibody staining was performed with antibodies against rhodopsin and LC3, an autophagosomal marker. Under identical conditions, the rapamycin group showed a higher number of foci with higher fluorescence intensity than was observed in the vehicle group (Fig. 7). This result suggests that the autophagy is promoted by rapamycin. When images of rhodopsin staining and LC3 staining were superimposed, the merged images showed more co-localization of rhodopsin and LC3 in the rapamycin group than in the vehicle group. These findings suggest that rhodopsin and LC3 are co-localized in photoreceptor cells.

**Fig. 8:**
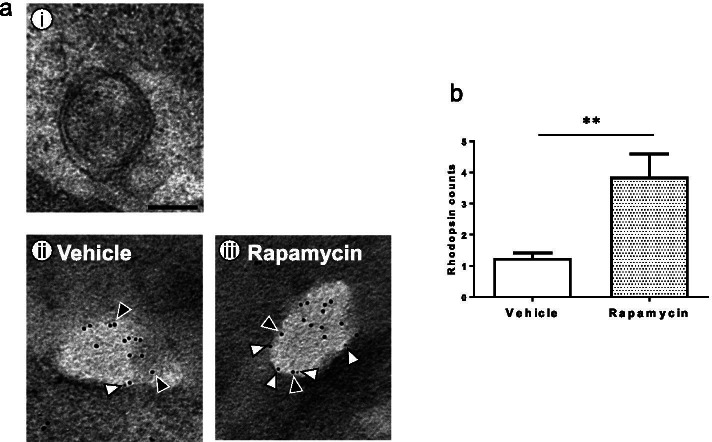
Analysis of retinas of P347L rats by immunoelectron microscopy. (a) Autophagosomes in retinas of P347L rats at the age of 10 days (i). Immunoelectron microscopy showing co-localization of rhodopsin labeled with gold colloid particles of 5 nm diameter, and LC3 labeled with gold colloid particles of 10 nm diameter, in the vesicles of vehicle group retina (ii) and rapamycin group retina (iii). Scale bar: 0.2 μm; white arrowheads: rhodopsin; black arrowheads: LC3. (b) Rhodopsin counts: 5 nm gold colloid labeled particles in autophagosome area. Graph showing the number of particles in P347L rats with or without rapamycin. Unpaired *t* test: **p < 0.01

Electron microscopy showed typical autophagosomal morphology in the cytoplasm of P347L rat photoreceptor cells (Fig. 8a-i). Immunoelectron microscopy of LC3 labeled with gold colloid particles of 10 nm diameter and rhodopsin labeled with gold colloid particles of 5 nm diameter showed that LC3 was localized in the membranes of vesicles, whereas rhodopsin was localized inside the vesicles (Fig. 8a-ii, iii). These findings demonstrate the uptake of rhodopsin by vesicles. Interestingly, there was a high uptake of rhodopsin by vesicles in the rapamycin group (Fig. 8a-iii, b). Consistent with the imaging results of fluorescent antibody staining (Fig. 7f), the number of vesicles was increased in the rapamycin group relative to that in the vehicle group (data not shown).

## Discussion

Previous studies have reported that the onset of retinal degeneration takes at least 2 weeks in RP animal models with rhodopsin mutations [1, 13]. In the P347L rats generated in the current study, the ONL began to decrease at the age of 9 days (Fig. 1). Thus, the P347L rat is a highly unusual RP model, which exhibits rapid retinal degeneration soon after birth, even before the retina is completely formed. Therefore, P347L rats are ideal for rapid in vivo analysis of drug efficacy in treating RP.

Heterotopic cells were found in P347L rat retinas. These cells were positive for rhodopsin but negative for TUNEL staining, suggesting that these are photoreceptor cells that have not undergone apoptosis. Staining for α-E-catenin, a component of the outer limiting membrane, showed abnormalities in the structure of the outer limiting membrane (data not shown). It is possible that abnormalities of the outer limiting membrane are involved in dissociating photoreceptor cells from the retinal ONL.

Impaired protein function due to gene mutations is linked to the ER stress response; thus, RP induces photoreceptor cell death [14]. Immunostaining of the P347L rat retina using rhodopsin antibodies showed abnormal accumulation of rhodopsin in the ONL of these rats (Fig. 2). The C-terminus of rhodopsin has been reported to be involved in the transport of rhodopsin to the outer segment in the photoreceptor cytoplasm [5, 6]. The P347L-type mutation generated in this study is a C-terminal mutation, which replaces a proline residue with leucine at position 347 of a total 348 amino acids. This mutation blocks rhodopsin transport, resulting in abnormal accumulation in the cytoplasm (Fig. 2). Biochemical analysis of the effects of cytoplasmic accumulation of rhodopsin on the photoreceptor cells in P347L rats showed significantly higher mRNA expression levels of the ER stress markers CHOP and Bip, as well as the autophagy markers LC3 and Atg5, compared to those seen in wild-type rats (Fig. 3). These findings show that rhodopsin is not appropriately transported but instead accumulates in the cytoplasm. This stresses the photoreceptor cytoplasm and activates an intracellular homeostatic mechanism involving the ER stress response and associated autophagy.

To determine the effectiveness of P347L rats for drug screening and to identify therapeutic agents against RP, the effects of a minute dose of rapamycin administered intravitreally were assessed. The ONL was thicker and showed significantly lower apoptosis in the rapamycin group than in the non-rapamycin group, suggesting that rapamycin is a potential therapeutic agent against RP. Rapamycin, an mTOR-inhibitor, is used clinically as an immunosuppressant. mTOR has a wide range of in vivo activities; its inhibition by rapamycin has been reported to be neuroprotective [10]. Rapamycin suppresses the loss of the ONL of photoreceptor cells, although the mechanism of action is not fully understood. To investigate the relationships between rapamycin and apoptosis pathways, morphological changes were assessed following inhibition of caspase-3. As expected, administration of the inhibitor suppressed loss of the ONL (Fig. 6). Similarly, rapamycin suppressed loss of the ONL and significantly reduced the number of apoptotic cells, suggesting that rapamycin is also involved in the caspase-dependent apoptosis pathway and acts to protect photoreceptors.

As inhibition of mTOR by rapamycin promotes autophagy [12, 15], the role of rapamycin in autophagy in photoreceptor cells was investigated. LC3 expression was higher in the rapamycin group than in the non-rapamycin group and co-localized with rhodopsin (Fig. 7), suggesting the uptake of rhodopsin by autophagosomes. Immunoelectron microscopy confirmed rhodopsin uptake by autophagosomes and revealed an increase in the number of autophagosomes (Fig. 8). These findings suggest that rapamycin promotes autophagy and autophagosomal uptake of the rhodopsin that has accumulated abnormally in the cytoplasm, thereby alleviating stress and delaying photoreceptor cell death.

The P347L rats generated in this study showed rapid postnatal retinal degeneration, possibly due to an overexpression of rhodopsin, prolonged activation of phototransduction, or activation of mislocalized opsin [7, 16, 17]. Previously reported model animals harboring the proline-347 mutation, such as P347L rabbits [2], P347S mice [18], and P347S / L pigs [19, 20] took a much longer time to undergo retinal degeneration. Suppressing or delaying retinal degeneration in the P347L model may contribute to the establishment of a therapeutic method for RP, and P347L rats represent an ideal animal model for this purpose.

## Conclusions

We developed a rat model for studying RP with rhodopsin mutation P347L. In this RP model, the time to onset of retinal degeneration was less than that of previously reported RP models with other rhodopsin gene mutations, enabling quicker in vivo evaluation of drug efficacy. Administration of rapamycin delayed photoreceptor cell degeneration by approximately 1 day. This was attributed to rapamycin alleviating stress caused by the mutant rhodopsin, and a delay in caspase-dependent cell death of the photoreceptors. If this treatment of the rapid RP rat model can prevent exacerbation of the condition, it may have a role in effectively preventing retinal degeneration.

## Methods

All animal experiments were approved by the Institutional Animal Care and Use Committee of Chubu University (Permit Numbers #3010028, #3010029 at Chubu University) and were conducted in accordance with institutional guidelines.

### Semi-knock in procedure

Rhodopsin P347L rats were generated by pronuclear injection of the BAC transgenic construct into Wister rat embryos, as previously reported [2]. The rat rhodopsin BAC clone CH230-100G19 was selected from a CHO230 Brown Norway Rat BAC library by searching a rat BAC ends database at the National Center for Biotechnology Information (NCBI) and obtained from the BACPAC Resources Center at Children’s Hospital Oakland Research Institute (CHORI). The BAC end sequences indicated that the BAC clone contained the whole rat rhodopsin genomic sequence along with an additional stretch of approximately 100 kb at both the 5ʹ- and 3ʹ-ends (Supplemental Fig. 1a).

A rhodopsin P347L BAC transgenic construct harboring C-to-T transition in exon 5 of the rat rhodopsin gene locus was generated by BAC recombineering (Supplemental Fig. 1b). A point mutation was introduced to the rat rhodopsin BAC clone using a Red/ET Counter Selection BAC Modification Kit (Gene Bridges, Heidelberg, Germany). In brief, an *rpsL-neo* counter selection cassette flanked by both the left and right (40 nucleotides) homology arm sequences directly adjacent to the C-to-T transition site of rhodopsin gene was amplified by PCR. The amplified *rpsL-neo* counter selection cassette was inserted into the rhodopsin gene of the Rat BAC clone by Red/ET recombination. The subcloned exon 5 fragment of the rat rhodopsin gene was modified with a C-to-T transition at proline 347 and serial restriction sites (KpnI, PstI, and BglII) in the 3ʹ-untranslated region, and then subcloned in pGEM-T easy vector for sequencing. The *rpsL-neo* cassette inserted in the rhodopsin gene of the rat BAC clone was replaced by the modified exon 5 fragment with rpsL counter selection. BAC modification was verified by Southern blot analysis and sequencing. The rhodopsin P347L BAC transgenic was purified for microinjection with a modified procedure as previously described [21]. The BAC transgenic construct was extracted from 250 ml of *E. coli* culture using Nucleobond Plasmid Purification kit (MACHEREY–NAGEL, Duren, Germany). For purification, 10 μg of the BAC transgenic construct was linearized overnight with PI-SceI endonuclease (New England Biolabs, Massachusetts, USA) cleaving the unique site in the BACe3.6 vector sequence. The linearized BAC DNA was separated with pulsed field gel electrophoresis (PFGE) and extracted from the preparative pulsed field gel by electroelution. After dialysis against TE buffer containing 0.1 mM EDTA, aliquots were subjected to PFGE for size and quality control (Supplemental Fig. 1c). The BAC DNA concentration was adjusted to 1 ng/μl for microinjection. Aliquots of BAC DNA solution were stored at 4 °C until microinjection.

### Intraocular injection in newborn rat eyes

Injection of a drug into the eye of 8 day old rats was carried out under isoflurane anesthesia. The drug was dissolved in 5% polyethylene glycol 400 and 5% Tween 80 and a 1 nmol aliquot was injected per eye using an ITO 30G micro-syringe (ITO CORPORATION, Shizuoka, Japan).

### Retinal histology

Rat eyes were fixed in 4% formaldehyde for 3 h at 4 °C. After dehydration by gradually increasing the concentration of ethanol, the eyeball was vertically divided in two and embedded in paraffin, sectioned vertically through the optic nerve (superior-inferior), and stained with HE. The thickness of the ONL was observed.

### Immunohistochemistry

Sections of rat eyes were deparaffinized and hydrated for antigen retrieval in 0.01 M citrate phosphate buffer (pH 6.0) for 2 h at 60 °C, and permeabilized with 0.05% triton X-100 in 4% bovine serum albumin (BSA)-phosphate buffered saline (PBS) for 30 min. The tissue sections were double stained sequentially with anti-rhodopsin antibody (1:200, sc-56472: Santa Cruz Biotechnology, CA, USA) and anti-LC3 antibody (1:200, PM036: MEDICAL & BIOLOGICAL LABORATORIES, Nagoya, Japan) in Can Get Signal immunostain immunoreaction enhancer solution (TOYOBO, Osaka, Japan) for 1 h at room temperature. This was followed by incubation with secondary antibodies, Alexa Fluor 488-conjugated anti-mouse IgG and Alexa Fluor 555-conjugated anti-rabbit IgG (Molecular Probes, Eugene, Oregon, USA), for 30 min at room temperature. Subsequently, coverslips were mounted on the double-stained tissue sections using fluorescent mounting medium (Mountant PermaFluor, Thermo Fisher Scientific, Massachusetts, USA) and examined using a FLUOVIEW FV1000 (OLYMPUS, Tokyo, Japan) and an Axio Imager 2 (Carl Zeiss, Oberkochen, Germany).

### TUNEL staining

TUNEL staining was carried out using a TACS-XL in situ Apoptosis Detection Kit according to the manufacturer’s instructions (Trevigen, Gaithersburg, Maryland, USA). Briefly, 4 μm thick paraffin sections were deparaffinized and rehydrated followed by sequential treatment with proteinase K and 3% hydrogen peroxide/methanol mixtures to inactivate endogenous peroxidase. Subsequently, the samples were incubated for 30 min at 37 °C with Anti-Brd U antibodies and apoptotic cells were detected using 3,3ʹ-diaminobenzidine (DAB) reaction kit (DAB Peroxidase Substrate Kit, ImmPACT, Vector Laboratories, California, USA).

### Electron microscopy

For transmission electron microscopy, rat eyes were fixed with glutaraldehyde, post-fixed with osmium tetroxide, dehydrated in a graded ethanol series, and embedded in Epon, as previously described [22]. Thin sections were stained with 5% uranyl acetate in 50% ethanol followed by 0.4% lead citrate and examined under a JEM-2100F electron microscope (JEOL, Tokyo, Japan) at 100 kV.

### Electron microscopic immunocytochemistry

Rat eyes were fixed with 1% glutaraldehyde and 4% paraformaldehyde in 100 mM HEPES (pH 7.4). The fixed eyes were dehydrated through an ascending series of ethanol up to 95%, and then soaked in a mixture of Lowicryl K4M resin (Polysciences Inc. Warrington, Pennsylvania, USA) and 95% ethanol for 1 h. Specimens were transferred into fresh Lowicryl K4M resin and kept in a refrigerator overnight. Rat eyes were then transferred into gelatin capsules filled with fresh Lowicryl K4M resin and polymerized under UV light for 2 weeks. Thin sections were obtained in the conventional way and mounted on 200-nickel-grids coated with formval. In brief, the sections were blocked with 4% BSA for 10 min and incubated with anti-rhodopsin antibody or anti-LC3 antibody in PBS containing 1% BSA for 2 h, rinsed 7 times with PBS, and then incubated with 10 or 15 nm gold-conjugated goat anti-rabbit or -mouse IgG (Amersham, Uppsala, Sweden) at a dilution of 1:30 in 1% BSA/ Tris-buffered saline for 1 h. After washing 8 times with PBS (1 min each), grids were post-fixed in 2% glutaraldehyde for 20 min and washed well with distilled water. All sections were stained with 5% uranyl acetate in distilled water prior to observation.

### Copy number analysis

Copy number analysis was carried out using the TaqMan® Copy number assay. Genomic DNA was isolated with the High Pure PCR Template Preparation Kit (Roche, Mannheim, Germany). The TaqMan® Copy number assay was performed using LightCycler TaqMan® Master (Roche, Mannheim, Germany) and the primer sets listed in Supplemental Table 1. Reactions were performed and analyzed on a LightCycler DX400 (Roche, Mannheim, Germany).

### qRT-PCR

Total RNA was isolated with the RNeasy Micro Kit (Qiagen, Hilden, Germany). First-strand cDNA was prepared from 1 μg of RNA using the PrimeScript™ RT reagent Kit (TaKaRa Bio Inc., Otsu, Japan). qRT-PCR was performed using LightCycler FastStart DNA MasterPLUS SYBR Green I (Roche, Mannheim, Germany) and the primer sets listed in Supplemental Table 1. Reactions were performed and analyzed on a LightCycler DX400 (Roche, Mannheim, Germany).

### Drugs

Rapamycin was purchased from Tokyo Chemical Industry Co. (Tokyo, Japan). Z-VAD-FMK, a pan-caspase inhibitor, was purchased from Medical & Biological Laboratories Co. (Nagoya, Japan).

### Statistical analysis

Statistical significance was analyzed by the *t*-test or two-way analysis of variance (ANOVA) with the Sidak’s multiple comparison test or one-way ANOVA with Tukey’s multiple comparison test. All analyses were performed using Prism 7 software (GraphPad; La Jolla, California, USA). *p* < 0.05 was regarded as significant.

## Supplementary Information

**Additional file 1.**

## Data Availability

Not applicable.

## Abbreviations

RP: Retinitis pigmentosa
ER: Endoplasmic reticulum
mTOR: Mammalian target of rapamycin
BAC: Bacterial artificial chromosome
ONL: Outer nuclear layer
DAB: 3, 3ʹ-Diaminobenzidine
BSA: Bovine serum albumin
qRT-PCR: Quantitative reverse-transcription polymerase chain reaction
HE: Hematoxylin eosin

## References

[1] Dryja TP, McGee TL, Hahn LB, Cowley GS, Olsson JE, Reichel E (1990). Mutations within the rhodopsin gene in patients with autosomal dominant retinitis pigmentosa. N Engl J Med.

[2] Kondo M, Sakai T, Komeima K, Kurimoto Y, Ueno S, Nishizawa Y (2009). Generation of a transgenic rabbit model of retinal degeneration. Invest Ophthalmol Vis Sci.

[3] Hartong DT, Berson EL, Dryja TP (2006). Retinitis pigmentosa. Lancet.

[4] Palczewski K, Kumasaka T, Hori T, Behnke CA, Motoshima H, Fox BA (2000). Crystal structure of rhodopsin: A G protein-coupled receptor. Science.

[5] Sung CH, Makino C, Baylor D, Nathans J (1994). A rhodopsin gene mutation responsible for autosomal dominant retinitis pigmentosa results in a protein that is defective in localization to the photoreceptor outer segment. J Neurosci.

[6] Tam BM, Moritz OL, Hurd LB, Papermaster DS (2000). Identification of an outer segment targeting signal in the cooh terminus of rhodopsin using transgenic *Xenopus laevis*. J Cell Biol.

[7] Alfinito PD, Townes-Anderson E (2002). Activation of mislocalized opsin kills rod cells: A novel mechanism for rod cell death in retinal disease. Proc Natl Acad Sci USA.

[8] Athanasiou D, Aguila M, Bellingham J, Li W, McCulley C, Reeves PJ (2018). The molecular and cellular basis of rhodopsin retinitis pigmentosa reveals potential strategies for therapy. Prog Retin Eye Res.

[9] Arriola Apelo SI, Lamming DW (2016). Rapamycin: An inhibiTOR of aging emerges from the soil. J Gerontol A Biol Sci Med Sci.

[10] Li J, Kim SG, Blenis J (2014). Rapamycin: one drug, many effects. Cell Metab.

[11] Pan T, Kondo S, Zhu W, Xie W, Jankovic J, Le W (2008). Neuroprotection of rapamycin in lactacystin-induced neurodegeneration via autophagy enhancement. Neurobiol Dis.

[12] Yoshida T, Ozawa Y, Suzuki K, Yuki K, Ohyama M, Akamatsu W (2014). The use of induced pluripotent stem cells to reveal pathogenic gene mutations and explore treatments for retinitis pigmentosa. Mol Brain.

[13] Nakazawa M, Hara A, Ishiguro S. Optical coherence tomography of animal models of retinitis pigmentosa: from animal studies to clinical applications. BioMed Res Int. 2019;8276140. 10.1155/2019/8276140 PMC6875330 31781647

[14] Marigo V (2007). Programmed cell death in retinal degeneration: targeting apoptosis in photoreceptors as potential therapy for retinal degeneration. Cell Cycle.

[15] Sato M, Seki T, Konno A, Hirai H, Kurauchi Y, Hisatsune A (2019). Rapamycin activates mammalian microautophagy. J Pharmacol Sci.

[16] Olsson JE, Gordon JW, Pawlyk BS, Roof D, Hayes A, Molday RS (1992). Transgenic mice with a rhodopsin mutation (Pro23His): A mouse model of autosomal dominant retinitis pigmentosa. Neuron.

[17] Chen J, Makino CL, Peachey NS, Baylor DA, Simon MI (1995). Mechanisms of rhodopsin inactivation in vivo as revealed by a COOH-terminal truncation mutant. Science.

[18] Li T, Snyder WK, Olsson JE, Dryja TP (1996). Transgenic mice carrying the dominant rhodopsin mutation P347S: Evidence for defective vectorial transport of rhodopsin to the outer segments. Proc Natl Acad Sci USA.

[19] Petters RM, Alexander CA, Wells KD, Collins EB, Sommer JR, Blanton MR (1997). Genetically engineered large animal model for studying cone photoreceptor survival and degeneration in retinitis pigmentosa. Nat Biotechnol.

[20] Kraft TW, Allen DE, Petters RM, Hao Y, Peng YW, Wong F (2005). Altered light responses of single rod photoreceptors in transgenic pigs expressing P347L or P347S rhodopsin. Mol Vis.

[21] Abe K, Hazama M, Katoh H, Yamamura K, Suzuki M (2004). Establishment of an efficient BAC transgenesis protocol and its application to functional characterization of the mouse Brachyury locus. Exp Anim.

[22] Yamazaki A, Nishizawa Y, Matsuura I, Hayashi F, Usukura J, Bondarenko VA (2013). Microtubule-associated protein tau in bovine retinal photoreceptor rod outer segments: comparison with brain tau. Biochim Biophys Acta.

