# Supplementary material for: A rat model for retinitis pigmentosa with rapid retinal degeneration enables drug evaluation in vivo
Source: Biol Proced Online. 2021 Jun 4;23:11. doi: 10.1186/s12575-021-00150-y (PMC8176615; doi:10.1186/s12575-021-00150-y)
Supplement: Supplementary file 1 — Additional file 1. [file 12575_2021_150_MOESM1_ESM.pdf]

Supplemental Fig. 1

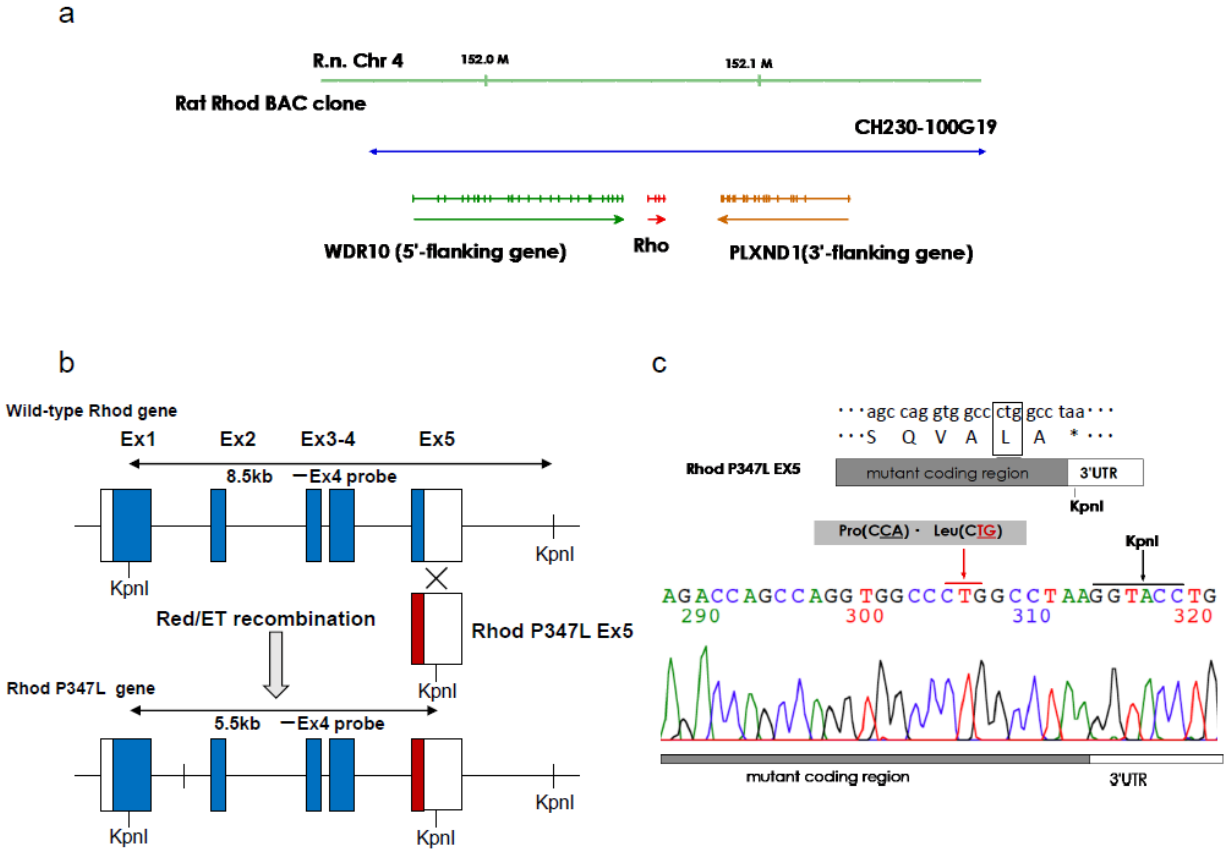

Supplemental Fig. 2

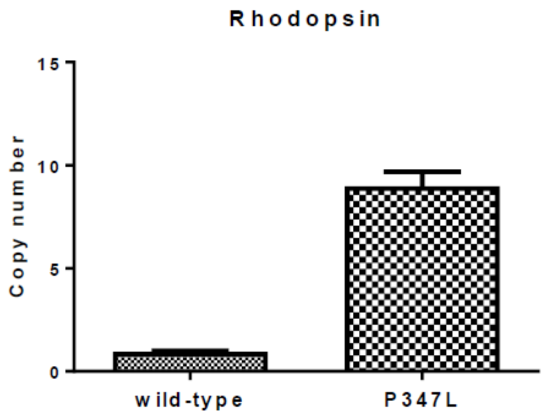

Supplemental Table 1

TaqMan® Copy Number Assays			
**Gene**	**Assay ID**	**Forward primers**	**Reverse primers**
Rho	Rho_CCI1MXB	5'-AGCAGGGAGAACGCTGAAC-3'	5'-GCCTTGGCTTAGCTGAGGATT-3'
Tfrc	Tfrc_CCKAK3J	5'-TGTCGCCCCTTTTCTTCTTTTAGAC-3'	5'-CACACTGGACTTCGCAACAC-3'

QRT-PCR		
**Gene**	**Forward primers**	**Reverse primers**
Rho	5'-GGCCTGTGGTCCCTGGTAGT-3'	5'-CCTCGGGGATGTACCTGGAC-3'
CHOP	5'-ACGAAGAGGAAGAATCAAAAACC-3'	5'-ACTTTCCTCTCATTCTCCTGCTC-3'
Bip	5'-CATCAATGAGCCAACAGCAG-3'	5'-TGATCAAAGTCTTCCCCACC-3'
LC3	5'-CCCGGTGATCATCGAGCGCT-3'	5'-CGGCGCCGGATGATCTTGAC-3'
Atg5	5'-GTTTGAATATGAAGGCACACCCC-3'	5'-TGCATTTCGTTGATCACCTGACT-3'
Rcvrn	5'-ACGTAGACGGCAATGGGACC-3'	5'-CCGCTTCTCTGGGGTGTTTT-3'
